# Effect of Inaudible Binaural Beats Stimulation Timing and Task Performance Level on Visuospatial Working Memory

**DOI:** 10.3390/bs16010076

**Published:** 2026-01-06

**Authors:** Kyu-Beom Kim, Min-Kyun Lee, Yong-Bin Jeong, Jeong-Min Kim, Mi-Hyun Choi, Hyung-Sik Kim, Byung-Chan Min, Soon-Cheol Chung

**Affiliations:** 1 Department of Biomedical Engineering, College of Science & Technology, Konkuk University, Chungju-si 27478, Republic of Korea; rlarbqja0507@kku.ac.kr (K.-B.K.); lmk9809@kku.ac.kr (M.-K.L.); veritas0831@kku.ac.kr (Y.-B.J.); wjdals6707@kku.ac.kr (J.-M.K.); kwjcc486@kku.ac.kr (M.-H.C.); 2 Department of Mechatronics Engineering, College of Science & Technology, Konkuk University, Chungju-si 27478, Republic of Korea; hskim98@kku.ac.kr; 3 Department of Industrial & Management Engineering, Hanbat National University, Daejeon 34158, Republic of Korea; bcmin@hanbat.ac.kr

**Keywords:** inaudible binaural beats, visuospatial working memory performance, stimulation timing, task performance level

## Abstract

This study aimed to investigate the effect of inaudible-frequency binaural beats (BB), excluding the influence of audible sound, on visuospatial working memory performance (VSWMP). In particular, the effects were examined in relation to the stimulation timing of the stimulus and the task performance level of participants. Thirty adults in their 20 s (20 males, 25.7 ± 1.8 years; 10 females, 24.3 ± 1.6 years) participated in the experiment. A 10 Hz BB stimulus was generated by simultaneously presenting 18,000 Hz and 18,010 Hz tones to the left and right ears, respectively. The experiment employed a within-participant design consisting of a rest phase (5 min) and a task phase (5 min), with four BB stimulation conditions: Control (no BB), Exp1 (BB during both rest and task phases), Exp2 (BB during rest only), and Exp3 (BB during task only). VSWMP was assessed using corrected hit rate and reaction time in a 3-back task. Results indicated that all BB conditions (Exp1, Exp2, Exp3) significantly improved VSWMP compared to the Control condition, regardless of the stimulation timing. When participants were grouped based on task performance level into high- and low-performing groups (HPG, LPG), significant improvements in VSWMP were particularly evident in the LPG across all BB conditions compared to the Control. Notably, in Exp3, LPG participants demonstrated VSWMP comparable to that of the HPG. In conclusion, while BB stimulation enhances VSWMP regardless of its stimulation timing, its effectiveness may vary depending on the task performance level.

## 1. Introduction

Brainwave entrainment (BWE) via binaural beats (BB) refers to the phenomenon wherein brainwave frequencies synchronize with the difference between two slightly different tones presented separately to each ear. For example, when a 400 Hz tone is presented to one ear and a 410 Hz tone to the other, the brain synchronizes to the difference frequency of 10 Hz (Gao et al., 2014; Vernon et al., 2014; Norhazman et al., 2012; Chaieb et al., 2015). Such BB-based BWE phenomena have been consistently observed across various frequency bands and have been physiologically validated under experimental conditions (Pratt et al., 2009; Jirakittayakorn & Wongsawat, 2017; Schwarz & Taylor, 2005; Draganova et al., 2008). For instance, Karino et al. (2006) reported entrainment effects in response to delta and theta BB stimuli, while Puzi et al. (2013) observed increased alpha and decreased beta activity in response to 10 Hz BB stimulation.

Based on this BWE mechanism, numerous studies have explored the potential of BB to enhance cognitive functions such as memory and attention (Wahbeh et al., 2007; Ingendoh et al., 2023; Garcia-Argibay et al., 2019; Engelbregt et al., 2019; Park et al., 2018; Mujib et al., 2021). Beauchene et al. (2016, 2017) reported that 15 Hz BB stimulation during task execution improved both visuospatial and verbal working memory performance. Kraus and Porubanová (2015) found that presenting 9.55 Hz alpha BB prior to task improved scores on an operation span task. Similarly, Rakhshan et al. (2022) reported that 10 Hz alpha BB stimulation led to slight performance improvements only in the visuospatial modality. However, other studies have reported no effect or even negative effects of BB on cognitive performance (Ahmadi et al., 2021; Leistiko et al., 2024). For example, Beauchene et al. (2016) reported that theta (5 Hz) and alpha (10 Hz) BBs presented during task actually decreased accuracy in visuospatial working memory tasks. Crespo et al. (2013) found that presenting beta (16 Hz) BB prior to task had no significant cognitive effects.

Although alpha-band activity (8–13 Hz) has been consistently linked to attention and working-memory processes (Jensen et al., 2002; Tuladhar et al., 2007; Van Dijk et al., 2010; Wang et al., 2017), the behavioral effects of alpha BB stimulation remain inconsistent across studies—some reporting improvements, while others showing null or even negative results. These inconsistencies highlight that the cognitive effects of BB have not yet been fully elucidated, suggesting that additional methodological factors may underlie the variability observed across studies. Various factors may contribute to the lack of consistent findings. In our previous study, we hypothesized that the perceived sound component of BB within the audible frequency range could influence cognitive processing. Most prior research employed BB within the audible range, where the presence of perceptible sound might confound the pure effects of BB itself. To address this, we employed BB stimuli in the inaudible frequency range—specifically using a 10 Hz BB generated by presenting 18,000 Hz and 18,010 Hz tones to each ear. Our results demonstrated that even inaudible BB stimulation led to a significant increase in alpha power and enhanced cognitive performance. These findings support the effectiveness of BB stimulation by minimizing the confounding influence of audible sound (Y. J. Kim et al., 2023; J. S. Kim et al., 2024).

Furthermore, the effects of BB stimulation may vary depending on the mode of stimulus presentation and participant characteristics. In previous studies, the timing of BB stimulation (e.g., before or during task execution) differed across experiments, which may have contributed to inconsistent findings. Additionally, although the effect of BB stimulation may differ depending on task performance level, few studies have accounted for this factor. In fact, research on cognitive enhancement using external stimuli has reported that enhancement effects tend to be more pronounced in groups with lower baseline performance levels (Reedijk et al., 2015; Mou et al., 2023).

To draw more scientifically robust conclusions about BB, further systematic investigations are required. Building on our previous work, which demonstrated BWE effects using inaudible BB stimulation, the present study aims to investigate whether the effectiveness of BB differs according to stimulation timing and participant characteristics. Specifically, we examine whether BB-induced cognitive enhancement varies depending on when the stimulus is presented (before task, during task, or both), and how these effects differ between high- and low-performing groups (HPG and LPG).

## 2. Methods

### 2.1. Participants

Thirty healthy adults in their 20s (male and female) with no reported hearing or cognitive impairments voluntarily participated in the experiment after responding to a recruitment notice (Table 1). All participants were fully informed about the experimental procedures and provided written informed consent prior to participation. They received monetary compensation for their involvement. This study was approved by the Institutional Review Board of Konkuk University (7001355-202105-HR-439).

### 2.2. Binaural Beats Stimulation

**Figure 1:**
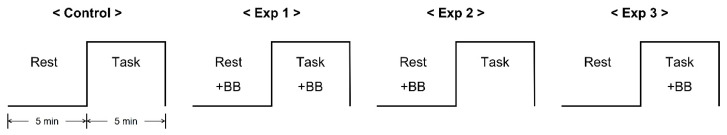
Experimental design.

To deliver a 10 Hz alpha-band BB stimulus, auditory tones of 18,000 Hz and 18,010 Hz were presented to the left and right ears, respectively, using an auditory stimulator (Model Q, Company G). The experimental procedure consisted of a rest phase (5 min) followed by a task phase (5 min). To examine the effects of BB stimulation timing, four conditions were implemented (Figure 1). In the Control condition, no BB stimulus was presented during either phase. In Experiment 1 (Exp1), BB stimulation was applied during both the rest and task phases. In Experiment 2 (Exp2), BB was presented only during the rest phase, while in Experiment 3 (Exp3), BB was presented only during the task phase. The Control condition was always presented first, and the order of the experimental conditions (Exp1–3) was counterbalanced to minimize order effects. To prevent carry-over effects, a 20 min rest period was provided between conditions. All participants completed all four conditions.

### 2.3. Visuospatial Working Memory Task

**Figure 2:**
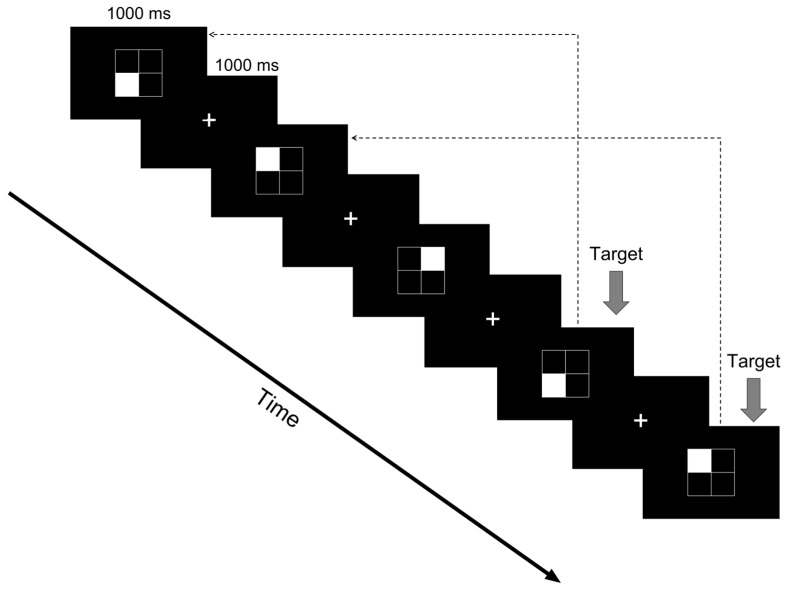
Procedure of the 3-back task used to assess VSWMP. A white fixation cross (“+”) was presented between stimuli to maintain attention. The white quadrant in each square indicates the highlighted (active) position.

To assess visuospatial working memory performance (VSWMP), a 3-back task was employed. This task has been validated in prior studies as a reliable measure for evaluating the cognitive effects of BB stimulation (Y. J. Kim et al., 2023; J. S. Kim et al., 2024). As illustrated in Figure 2, the visual stimuli consisted of squares divided into four quadrants, with one randomly highlighted in white. Participants were instructed to press a button (the space bar on the keyboard) as quickly as possible whenever the position of the current stimulus matched that of the stimulus presented three trials earlier. A total of 150 stimuli were presented, including 50 target trials. Each stimulus was displayed for 1 s, followed by a white fixation cross (+) shown for 1 s to help maintain attention. Prior to the experimental trials, participants completed a practice session to ensure full understanding of the task procedure.

### 2.4. Behavioral Performance Measurement and Analysis

VSWMP under different BB stimulation timings was evaluated based on corrected hit rate and reaction time. The corrected hit rate was defined as the difference between the hit rate (TP/(TP + FN)) and the false alarm rate (FP/(FP + TN)), where TP, FP, FN, and TN denote true positives, false positives, false negatives, and true negatives, respectively. Reaction time was defined as the elapsed time between the presentation of a target stimulus and the participant’s button press. A repeated-measures ANOVA (RM-ANOVA) was conducted to analyze differences in VSWMP across the four conditions. When a significant main effect was found, Bonferroni-corrected post hoc tests were conducted (SPSS Statistics 29, IBM, Armonk, NY, USA).

To examine the effect of BB stimulation based on task performance level, participants were divided into two groups (HPG and LPG) using the average corrected hit rate in the Control condition (0.55) as the cutoff. Each group consisted of 15 participants (Male: 10, Female: 5). The mean corrected hit rate of the HPG was 0.70 ± 0.09, while that of the LPG was 0.39 ± 0.09. A mixed-design ANOVA was conducted to analyze differences in VSWMP across conditions (within-subjects factor) and groups (between-subjects factor). When an interaction effect was significant, simple effects analyses were subsequently performed.

## 3. Results

### 3.1. Effects of BB Stimulation Timing on Corrected Hit Rate and Reaction Time

**Figure 3:**
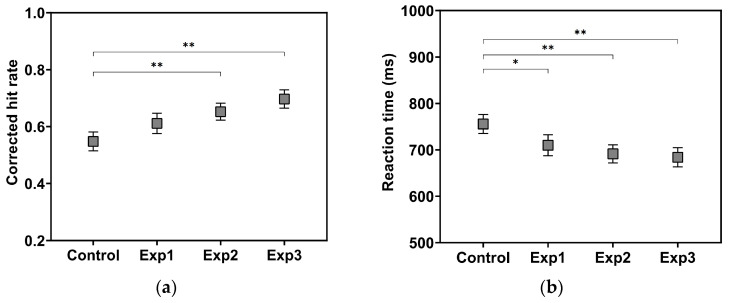
Comparison of VSWMP across BB stimulation conditions. (**a**) Show the corrected hit rate for each condition. (**b**) Shows the reaction time for each condition. Error bars indicate standard errors (* *p* < 0.05, ** *p* < 0.01).

RM-ANOVA results revealed a significant main effect of condition on corrected hit rate (*F*(3, 87) = 6.91, *p* < 0.001, *η*^2^ = 0.19) (Table 2). Bonferroni-corrected pairwise comparisons (Figure 3a) showed that corrected hit rate in Exp2 (*t*(29) = −3.90, *p* = 0.003, *d* = 0.71) and Exp3 (*t*(29) = −3.47, *p* = 0.010, *d* = 0.63) was significantly higher than in the Control condition.

Reaction time analysis likewise yielded a significant main effect of condition (*F*(3, 87) = 10.05, *p* < 0.001, *η*^2^ = 0.26) (Table 2). As shown in Figure 3b, reaction time in Exp1 (*t*(29) = 2.91, *p* = 0.047, *d* = 0.53), Exp2 (*t*(29) = 3.98, *p* = 0.003, *d* = 0.73), and Exp3 (*t*(29) = 5.90, *p* < 0.001, *d* = 1.08) was significantly shorter than in the Control condition. All pairwise comparisons, including non-significant results, are reported in Table 3 for completeness.

### 3.2. Effects of BB Stimulation Timing and Task Performance Level on Corrected Hit Rate and Reaction Time

**Figure 4:**
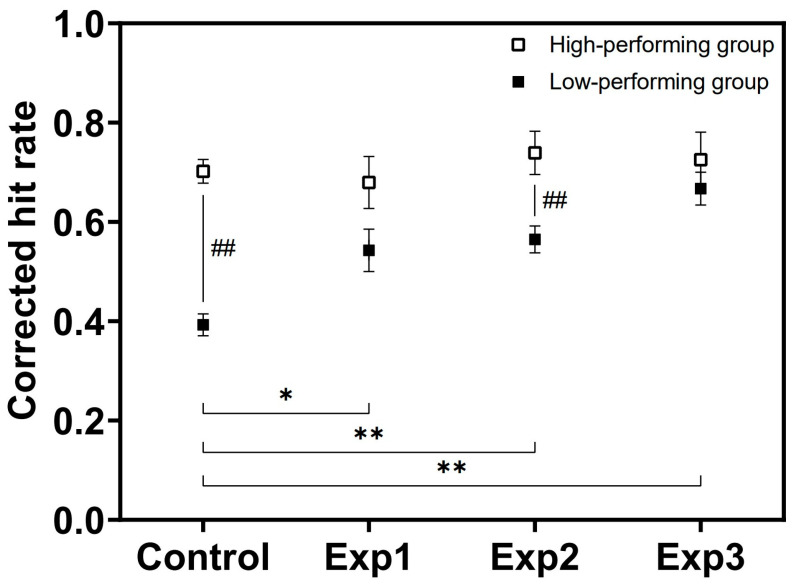
Comparison of corrected hit rate across BB stimulation conditions and groups. Asterisks (*) indicate significant differences between conditions within each group, while hash symbols (#) indicate significant differences between groups within each condition (*: *p* < 0.05, ** and ##: *p* < 0.01). Error bars indicate standard errors.

A mixed-design ANOVA on corrected hit rate revealed a significant interaction between condition and group (*F*(3, 84) = 5.45, *p* = 0.002, *η*^2^ = 0.16) (Table 4). As shown in Figure 4, simple effect analysis indicated that within-group comparisons across conditions revealed significant differences only in the LPG. Specifically, the corrected hit rate in the LPG was significantly higher in Exp1 (*t*(14) = −3.28, *p* = 0.017, *d* = 0.94), Exp2 (*t*(14) = −5.05, *p* < 0.001, *d* = 1.47), and Exp3 (*t*(14) = −5.28, *p* < 0.001, *d* = 1.97) compared to the Control condition. For the HPG, no significant differences were observed across conditions (Table 5). Between-group comparisons for each condition revealed that the LPG showed significantly lower corrected hit rates than the HPG in the Control (*t*(28) = 9.45, *p* < 0.001, *d* = 3.45), and Exp2 (*t*(28) = 3.39, *p* = 0.002, *d* = 1.24) conditions.

**Figure 5:**
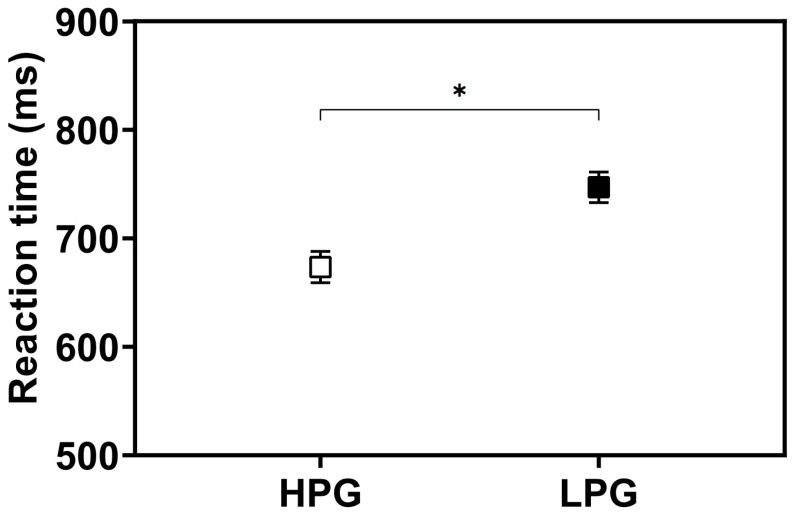
Comparison of reaction time between groups (*: *p* < 0.05). Error bars indicate standard errors.

A mixed-design ANOVA on reaction time revealed no significant interaction between condition and group (*F*(3, 84) = 0.33, *p* = 0.803, *η*^2^ = 0.01) (Table 4). However, the main effects of both condition (*F*(3, 84) = 9.82, *p* < 0.001, *η*^2^ = 0.26) and group (*F*(1, 28) = 4.25, *p* = 0.049, *η*^2^ = 0.13) were statistically significant (Table 4). The average changes in reaction time across conditions were consistent with those shown in Figure 3b. As no interaction was observed, the main effect of condition was interpreted independently of group (collapsed across groups). Post hoc analysis indicated that reaction time was significantly reduced in Exp1 (*t*(29) = 2.91, *p* = 0.047, *d* = 0.53), Exp2 (*t*(29) = 3.98, *p* = 0.003, *d* = 0.73), and Exp3 (*t*(29) = 5.90, *p* < 0.001, *d* = 1.08) (Table 3) compared to the Control condition. Regarding the main effect of group, post hoc analysis revealed that the LPG exhibited significantly longer reaction times than the HPG, as shown in Figure 5 (*t*(28) = 2.06, *p* = 0.049, *d* = 0.75).

## 4. Discussion

This study investigated the effects of inaudible alpha BB stimulation on VSWMP, focusing on the timing of BB stimulation and task performance level. Compared to the Control condition, all BB stimulation conditions (Exp1, Exp2, Exp3) led to significant improvements in VSWMP. However, there were no significant differences in VSWMP between the three BB stimulation timing conditions. Group-wise analysis based on task performance revealed that significant enhancements in VSWMP were particularly evident only in the LPG across all BB conditions compared to Control. Notably, in the condition where BB was presented during the task phase (Exp3), the LPG exhibited VSWMP comparable to that of the HPG.

BB stimulation improved VSWMP regardless of the stimulation timing. In our previous study, we reported that presenting inaudible alpha BB during task execution induced alpha entrainment in the central, parietal, temporal, and occipital brain regions, as well as activation in the frontal and right parietal areas, which contributed to enhanced VSWMP (Y. J. Kim et al., 2023; J. S. Kim et al., 2024). In the present study, the condition in which BB was presented during the task phase (Exp3) likely induced sufficient brainwave entrainment throughout task execution, thereby positively influencing performance. The observed improvement in VSWMP when BB was presented prior to the task (Exp2) may be attributed to an after-effect, wherein the BB-induced brain activity persisted into the task phase and continued to support cognitive performance. Although Exp1 was expected to show the strongest improvement by combining both effects, its performance gain was relatively weaker. One possible explanation is neural habituation, a phenomenon widely reported in cognitive neuroscience, caused by continuous exposure to BB across both rest and task phases, which may have reduced the efficiency of task-related entrainment (Thompson & Spencer, 1966; Groves & Thompson, 1970; Schmid et al., 2015). However, as this interpretation remains speculative, further neurophysiological investigation is warranted to verify this possibility. Collectively, considering that no statistically significant differences were observed among the different BB stimulation timings, and that the observed effect sizes between experimental conditions were all below the medium threshold (Cohen’s *d* < 0.5), it is likely that the presence or absence of BB stimulation itself served as the primary factor influencing cognitive enhancement, rather than the specific timing of its presentation.

The comparison between groups based on task performance level revealed that the cognitive enhancement effect of BB stimulation was more pronounced in the LPG. This phenomenon can be interpreted from the perspective of baseline dependency, which posits that external stimulation tends to have a greater effect on individuals with lower initial performance levels. Previous studies have similarly reported that individuals with lower abilities tend to show greater improvements following external stimulation or training interventions (Reedijk et al., 2015; Mou et al., 2023; Shapira et al., 2021; Pileckyte & Soto-Faraco, 2024). For example, Reedijk et al. (2013) investigated the effects of alpha BB on divergent thinking, using spontaneous eye blink rates (EBR) as an indicator to classify individuals’ cognitive baseline levels. The results showed that BB stimulation enhanced performance on the alternate uses task—a measure of divergent thinking—particularly in individuals with low EBR, while no such improvement was observed in those with high EBR. These findings suggest that BB stimulation may be particularly effective for enhancing cognitive performance in lower-performing groups.

Moreover, while the overall analysis without group separation showed similar levels of VSWMP across all BB conditions, the group-based analysis revealed that in the LPG, the effect of BB was most pronounced in the Exp3 condition (BB stimulation presented only during task execution). In this condition, no significant group difference was observed and the effect size was small, suggesting that both groups performed at comparable levels. Of note, Exp1 also showed no statistically significant group difference; however, the effect size was large (*d* = 0.74), and the *p*-value was slightly above the conventional threshold (*p* = 0.053). Considering these results, it may be inappropriate to conclude that the two groups performed equivalently in this condition. Although further verification is needed, this finding suggests that the effectiveness of BB stimulation, particularly with respect to its stimulation timing, may vary depending on cognitive characteristics.

This study employed BB in the inaudible frequency range to isolate and examine the pure effects of BB stimulation, independent of audible sound. The results demonstrated that BB stimulation positively influenced cognitive performance regardless of the stimulation timing. However, in the LPG, the degree of improvement varied depending on the timing of BB stimulation. These findings suggest that inconsistent results in previous BB studies may, in part, be attributed to a lack of consideration for individual differences such as baseline task performance. In other words, while BB stimulation can indeed enhance cognitive function, its effects may manifest differently depending on participant characteristics, and such variability should be taken into account. Nevertheless, this study assessed only short-term effects; thus, further research is needed to investigate the long-term sustainability of BB-induced enhancement following repeated exposure. In addition, since no physiological measures such as EEG were used to verify actual brainwave entrainment, future studies should aim to elucidate the neurophysiological mechanisms of BB stimulation as a function of stimulation timing and task performance level.

## Figures and Tables

**Table 1:** Information about participants.

	Number of Participants	Mean of Age (SD)
Male	20	25.7 (±1.8)
Female	10	24.3 (±1.6)
Total	30	25.2 (±1.8)

SD: Standard deviation.

**Table 2:** Results of repeated-measures ANOVA across all participants for corrected hit rate and reaction time.

	*SS*	*F*(3, 87)	*p*	*η* ^2^
Corrected hit rate	0.36	6.91	<0.001 **	0.19
Reaction time	93,497.07	10.05	<0.001 **	0.26

SS: Type 3 Sum of Square, *η*^2^: Partial Eta-Squared, **: *p* < 0.01.

**Table 3:** Post hoc comparisons across all participants for corrected hit rate and reaction time.

	Comparison	*MD*	*t*(29)	*p*	*d*
Corrected hit rate	Control–Exp1	−0.06	−1.80	0.499	0.33
	Control–Exp2	−0.10	−3.90	0.003 **	0.71
	Control–Exp3	−0.15	−3.47	0.010 **	0.63
	Exp1–Exp2	−0.04	−1.52	0.831	0.28
	Exp1–Exp3	−0.09	−2.44	0.125	0.45
	Exp2–Exp3	−0.04	−1.28	1.000	0.24
Reaction time	Control–Exp1	45.74	2.91	0.047 *	0.53
	Control–Exp2	64.37	3.98	0.003 **	0.73
	Control–Exp3	71.77	5.90	<0.001 **	1.08
	Exp1–Exp2	18.63	1.24	1.000	0.23
	Exp1–Exp3	26.03	1.87	0.463	0.34
	Exp2–Exp3	7.40	0.58	1.000	0.11

MD: Mean Difference, *d*: Cohen’s d, *: *p* < 0.05, **: *p* < 0.01.

**Table 4:** Results of mixed-design ANOVA for corrected hit rate and reaction time across condition and group.

	Source	*SS*	*F*(df1, df2)	*p*	*η* ^2^
Corrected hit rate	Condition	0.36	*F*(3, 84) = 7.97	<0.001 **	0.22
	Group	0.86	*F*(1, 28) = 17.84	<0.001 **	0.39
	Condition × Group	0.25	*F*(3, 84) = 5.45	0.002 **	0.16
Reaction time	Condition	93,497.07	*F*(3, 84) = 9.82	<0.001 **	0.26
	Group	162,116.01	*F*(1, 28) = 4.25	0.049 *	0.13
	Condition × Group	3147.00	*F*(3, 84) = 0.33	0.803	0.01

SS: Type 3 Sum of Square, *df*: Degree of Freedom, *η*^2^: Partial Eta-Squared, *: *p* < 0.05, **: *p* < 0.01.

**Table 5:** Post hoc comparisons for corrected hit rate following the significant interaction between condition and group, including both within-group and between-group contrasts.

Type	Comparison	*MD*	*t*(14)	*p*	*d*
Within-group (HPG)	Control–Exp1	0.02	0.49	1.000	0.12
	Control–Exp2	−0.04	−1.08	1.000	0.25
	Control–Exp3	−0.02	−0.46	1.000	0.10
	Exp1–Exp2	−0.06	−1.54	0.803	0.55
	Exp1–Exp3	−0.05	−0.94	1.000	0.21
	Exp2–Exp3	0.01	0.28	1.000	0.06
Within-group (LPG)	Control–Exp1	−0.15	−3.28	0.017 *	0.94
	Control–Exp2	−0.17	−5.05	<0.001 **	1.47
	Control–Exp3	−0.27	−5.28	<0.001 **	1.98
	Exp1–Exp2	−0.02	−0.59	1.000	0.13
	Exp1–Exp3	−0.12	−2.53	0.103	0.81
	Exp2–Exp3	−0.10	−2.15	0.244	0.73
**Type**	**Comparison**	***MD***	***t*** **(28)**	***p***	***d***
Between-group	Control (HPG–LPG)	0.31	9.45	<0.001 **	3.45
	Exp 1 (HPG–LPG)	0.14	2.02	0.053	0.74
	Exp 2 (HPG–LPG)	0.17	3.39	0.002 **	1.24
	Exp 3 (HPG–LPG)	0.06	0.92	0.367	0.33

MD: Mean Difference, *d*: Cohen’s d, *: *p* < 0.05, **: *p* < 0.01.

## Data Availability

The data presented in this study are available on request from the corresponding author.

## Abbreviations

The following abbreviations are used in this manuscript:

BB	Binaural beats
VSWMP	Visuospatial working memory performance
HPG	High-performing group
LPG	Low-performing group
BWE	Brainwave entrainment
Exp	Experiment
ANOVA	Analysis of variance
